# Towards single-cell ionomics: a novel micro-scaled method for multi-element analysis of nanogram-sized biological samples

**DOI:** 10.1186/s13007-020-00566-9

**Published:** 2020-03-06

**Authors:** Anle Chen, Thomas H. Hansen, Lene I. Olsen, Michael Palmgren, Søren Husted, Jan K. Schjoerring, Daniel Pergament Persson

**Affiliations:** Department of Plant and Environmental Sciences, Faculty of Science, University of Copenhagen, Thorvaldsensvej 40, 1871 Frederiksberg C, Denmark

**Keywords:** ICP-MS, Micro-scaled, Multi-elemental analysis, Plant tissue, Pressurized microwave digestion, *Arabidopsis thaliana* seeds, Laser capture microdissection (LCM), Barley (*Hordeum vulgare*)

## Abstract

**Background:**

To understand processes regulating nutrient homeostasis at the single-cell level there is a need for new methods that allow multi-element profiling of biological samples ultimately only available as isolated tissues or cells, typically in nanogram-sized samples. Apart from tissue isolation, the main challenges for such analyses are to obtain a complete and homogeneous digestion of each sample, to keep sample dilution at a minimum and to produce accurate and reproducible results. In particular, determining the weight of small samples becomes increasingly challenging when the sample amount decreases.

**Results:**

We developed a novel method for sampling, digestion and multi-element analysis of nanogram-sized plant tissue, along with strategies to quantify element concentrations in samples too small to be weighed. The method is based on tissue isolation by laser capture microdissection (LCM), followed by pressurized micro-digestion and ICP-MS analysis, the latter utilizing a stable µL min^−1^ sample aspiration system. The method allowed for isolation, digestion and analysis of micro-dissected tissues from barley roots with an estimated sample weight of only ~ 400 ng. In the collection and analysis steps, a number of contamination sources were identified. Following elimination of these sources, several elements, including magnesium (Mg), phosphorus (P), potassium (K) and manganese (Mn), could be quantified. By measuring the exact area and thickness of each of the micro-dissected tissues, their volume was calculated. Combined with an estimated sample density, the sample weights could subsequently be calculated and the fact that these samples were too small to be weighed could thereby be circumvented. The method was further documented by analysis of Arabidopsis seeds (~ 20 µg) as well as tissue fractions of such seeds (~ 10 µg).

**Conclusions:**

The presented method enables collection and multi-element analysis of small-sized biological samples, ranging down to the nanogram level. As such, the method paves the road for single cell and tissue-specific quantitative ionomics, which allow for future transcriptional, proteomic and metabolomic data to be correlated with ionomic profiles. Such analyses will deepen our understanding of how the elemental composition of plants is regulated, e.g. by transporter proteins and physical barriers (i.e. the Casparian strip and suberin lamellae in the root endodermis).

## Background

Because of differences in uptake, storage and transport, mineral elements are unevenly distributed throughout plant tissues, both at the organ, tissue, cellular and sub-cellular levels [1, 2]. This implies that many plant tissues/cells are functionally different from their neighbouring tissues and display cell-specific ionomic profiles [3]. Multi-element, or “ionomic” studies, which is a concept introduced more than 10 years ago, combines high-throughput element profiling of biological tissues with genetic mapping. In plants, this work was pioneered by Lahner et al. [4], who utilized high-throughput ICP-MS analyses of leaves in order to reveal ionomic and genetic connections in mutants of the model plant *Arabidopsis thaliana* [4]. Since then, a range of ionomic studies have been performed, including other plant species (e.g. rice, barley, soybean and tomato), and yeast [5]. The combination of genetics and ionomics has so far resulted in the identification of many genes controlling the ionome, at the whole plant level [3].

Multi-element analyses have traditionally mostly been carried out on dry, powdered material with a known weight per sample. Producers of certified reference materials typically recommend a minimum of 200 mg of homogenized sample material in order to gain a full, multi-element analysis with high data quality (i.e. high accuracy and precision). This renders many samples out of reach for element profiling/ionomics analyses.

Analytical procedures have been developed that can handle sample sizes considerably lower than 200 mg [6–9]. Samples of 1 mg of certified reference material were analysed with acceptable reproducibility (SE < 8%) and accuracies within ± 10% for 10 elements by Hansen et al. [9]. In this size range, ionomic profiling will be possible for individual plant organs (e.g. leaves, stems, roots and grains), but only very rarely at tissue or cellular levels, where samples will have µg or even ng weights. These small sample sizes are challenging not only because of the weak signals generated, but also by the risk of contamination and the need to perform a complete and uniform digestion prior to analysis. Moreover, it is difficult, in fact often impossible, to accurately determine the sample weight of such samples. In order to address these limitations, methods for single-cell ICP-MS (SC-ICP-MS) analysis have in recent years been developed for multi-element profiling at the cellular level, typically on isolated cell cultures [10, 11]. Single-cell ICP-MS is a technique that utilizes either pneumatic nebulization or micro-droplet generation of cell suspensions in order to introduce individual cells into the plasma of the ICP-MS. In SC-ICP-MS, absolute quantification is possible, typically reporting concentrations as the weight of element per cell [12]. The advancement of such technologies has enabled detailed characterization of cell-specific molecular profiles and has led to identification of genetic players involved in fine-tuning the regulation and distribution of the trace elements selenium (Se), copper (Cu), iron (Fe) and zinc (Zn) in human cell cultures [13].

If isolated cell cultures are not available, which is often the case in plant science, tissue-specific cell types from plants can be collected with fluorescence-activated cell sorting (FACS) of protoplasts for subsequent analysis with various techniques [14, 15]. With respect to ionomic profiling, the main challenge with this technique is the liquid environment in which the cell sorting is conducted, as it imposes a risk of ion leaking, especially for monovalent ions that do not form covalent bonds [e.g. sodium (Na^+^) and potassium (K^+^)]. Another challenge is the enzymatic degradation of the cell wall that needs to be performed, which may also induce leakage of ions and/or contamination from the enzymes. The technique requires that different cell types express specific markers to allow for the sorting to work, which to date is only available in mutants of Arabidopsis. The FACS approach to sample collection of plant material for ionomic studies is currently being developed, but to the best of our knowledge, no results have yet been published.

Tissues or single cells may also be collected by laser capture microdissection (LCM), utilizing laser cutting and catapulting for collection of regions of special interest in microscopic specimens. LCM is a microscope-based technique, which permits rapid separation and contact-free collection of desired cells or specific tissues [16, 17]. The LCM technique has been extensively used in combination with genomics, transcriptomics and proteomics analyses [18, 19], but only very rarely for multi-element analysis [20]. Samples can be either from heterogeneous mixtures of cell populations or from dry sections mounted on culture plates or membrane-covered glass slides. The tissue collection involves several steps: sample preparation, outlining and laser-cutting of the selected area and collection of the dissected cells or tissues by pressure catapulting against gravity into an adhesive cap. The LCM laser beam has a 1 µm diameter, allowing single cells as well as larger tissue sections to be collected. The challenges with ion leakage and displacement during cell sorting or analysis in a liquid environment (i.e. FACS and SC-ICP-MS) is not a concern with LCM, since sample collection is performed from frozen and freeze-dried samples, without addition of any liquids. However, preparing dry samples of good quality without an altered ion composition requires a different sample preparation compared to other omics techniques, but these challenges have already been addressed [21]. After digestion and dilution, the samples end up in an ICP-MS-compatible solution. Hence, all analytical benefits that has been developed for absolute quantification of liquid ICP-MS samples becomes available, including calibration standards, internal/external standards and the use of certified reference material for data validation.

Element profiling of small-sized samples raises special challenges with respect to sample digestion in order to avoid contamination and ensure data quality. Whereas earlier digestion methods were based either on open digestion at relatively low temperatures or closed microwave-assisted digestion systems with individual bombs that were pressurised upon heating, the most recent generation of commercially available microwave digestion facilities pressurization of the whole digestion chamber, using an inert gas [22, 23]. This strategy has several advantages. Firstly, the pressurized environment (> 40 bar) increases the boiling point of the digestion media (mixtures of nitric acid and hydrogen peroxide) to above 240 °C, ensuring a total and uniform digestion of all samples in the chamber, without boiling. Secondly, since the digestion is performed without boiling, large numbers of samples, also with different volumes, can be homogeneously digested in the same digestion chamber without the risk of cross contamination [23].

The overall objective of this study was to push the boundaries for ionomic studies of µg and ng-sized biological samples, which are not available as isolated cells or cell cultures. The first goal was to collect pure tissue samples and thereafter secure a complete digestion of these. Once digested, the next goal was to enable quantitative multi-elemental analysis of the resulting low volume samples (300 µL) with precision and accuracy comparable to state-of-the-art procedures. As an integral part of the study, various ways to determine the element concentrations per unit sample weight of samples that were too small to weigh accurately, were pursued.

Using the developed method, a number of elements were successfully quantified in certified reference material (CRM) from apple leaves, in seeds of Arabidopsis and in isolated barley root tissues. In the barley root tissue fractions, with estimated weights as low as 400 ng, K, Mn, P and Mg were all successfully quantified in two distinct tissue types (cortex and stele). We conclude that tissue isolation with LCM in combination with micro-scaled digestion and multi-element ICP-MS analysis is possible, paving the way for future ionomic analyses at the tissue or single-cell levels.

## Results

### Analysis of certified reference material (CRM)

**Fig. 1:**
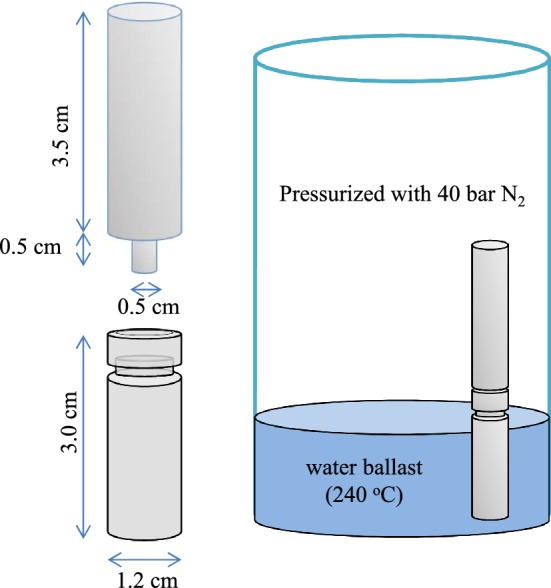
Design of the micro-scaled sample digestion system. Schematic figure showing the design of the micro-scaled sample digestion system, shown here with a 0.3 mL vial

For validation of the method, dried certified reference material (CRM) consisting of apple leaves were digested in the micro-scaled sample digestion system (Fig. 1) and then analysed in gradually decreasing sample amounts. The CRM was certified for all essential plant nutrients as well as a range of other elements of interest in plant science, including some beneficial elements (e.g. Na and Se) and toxic metals (e.g. Al, Cd and As). The true concentration values (accuracy) and their uncertainties (precision) were stated in the certificate and were based on analysis of a minimum of 200 mg sample. Using 2 mg (2000 µg) samples, we obtained accuracies between 96 and 103% of the reported values for all elements except B and Mo, which both gave a slightly lower value of 87% (Table 1). The accuracy decreased with about 10% for all elements except B and Zn, when decreasing the sample quantity from 2000 to 500 µg (Table 1). The precision of the certified element concentrations varied from 1% for Ca to 14% for Mo (Table 1, left). Our measurements had similar or, in case of e.g. Mn, B, Cu, Ni and Mo, even better precision than the certified values. The precision of the results for Mn and Cu did only increase slightly (1–4% and 2–3%, respectively) when the sample size decreased from 2000 to 500 µg (Table 1). The same was the case for elements such as K, Mg, Al and Ca present in relatively high concentrations. However, for Zn, B, Ni and Mo the precision became poorer with decreasing sample size, especially that of Ni, where the relative standard deviation (RSD) increased from 7% (2000 µg) to 62% (500 µg). A general decline in precision must be expected since the variability caused by uneven distribution of elements increases as the sample size decreases, as is also the case for sample weighing inaccuracies.

**Table 1:** Accuracy and precision of ICP-MS measurements of certified reference material (NIST 1515, apple leaf)

Element	Certified reference concentrations, µg g^−1^	Sample quantity, µg		
	(RSD %)	500	1000	2000
		Accuracy, % (precision, RSD in %)		
K	16,100 ± 200 (1)	89 (4)	97 (4)	100 (1)
Ca	15,260 ± 150 (1)	88 (4)	94 (4)	98 (2)
Mg	2710 ± 80 (3)	93 (4)	100 (4)	103 (3)
Al	286 ± 9 (3)	91 (4)	96 (7)	98 (1)
Mn	54 ± 1 (6)	87 (4)	93 (4)	96 (1)
B	27 ± 2 (7)	87 (11)	86 (11)	87 (5)
Zn	12.5 ± 0.3 (2)	100 (19)	101 (19)	98 (2)
Cu	5.64 ± 0.24 (4)	96 (3)	99 (3)	97 (2)
Ni	0.91 ± 0.12 (13)	128 (62)	102 (62)	98 (7)
Mo	0.094 ± 0.013 (14)	84 (32)	102 (32)	87 (7)

Accuracy and precision of ICP-MS measurements of certified reference material (NIST 1515, apple leaf), digested in quantities from 500 to 2000 µg. The relative amount of digestion media and the following dilution was kept constant. The accuracy is calculated as the percentage of the certified reference concentration and the precision as the relative standard deviation in percent (n = 8)

### Analysis of *Arabidopsis thaliana* seeds

One single Arabidopsis seed weighs approximately 20 µg, which is too low to be accurately weighed. One seed is, however, not impossible to digest and analyse. To test if reliable results could be generated from such a small sample size, we analysed and compared seed-batches of 100 mg (corresponding to ≈ 5000 seeds), 1 mg (≈ 50 seeds), 10 seeds (corresponding to ≈ 200 µg) and one single seed (≈ 20 µg). In order to be able to compare the element concentrations in individual seeds across the four batches (Table 2), an average seed weight of 19.1 µg was used for calculation of the element concentration per seed. In the samples consisting of 10 seeds, none of the investigated elements deviated significantly from the results obtained when 100 mg was analysed (Table 2). Thus, reliable and reproducible results could be generated from a 10 seed batch with a sample size of only ~ 200 µg. In the single seed analysis, the concentration of five out the 10 tested elements were statistically different relative to the 100 mg batch samples (Table 2). Obviously, a single seed analysis is much more sensitive to inter-seed variability than a batch of seeds.

**Table 2:** Dry weight concentrations in wild type *Arabidopsis thaliana*

Element	100 mg seed, n = 4		1 mg seed, n = 4		10 seeds, n = 10		1 seed, n = 10		
	%g g^−1^	RSD%	%g g^−1^	RSD%	%g g^−1^	RSD%	%g g^−1^	RSD%	
S	1.60 ± 0.06	4	1.32 ± 0.18	13	1.71 ± 0.25	15	1.79 ± 0.80	45	P = 0.287*
K	1.55 ± 0.05	4	1.41 ± 0.21	15	1.89 ± 0.21	11	2.25 ± 0.60	27	P = 0.07*
P	1.16 ± 0.04^ab^	3	0.99 ± 0.131^a^	13	1.32 ± 0.17^ab^	13	1.49 ± 0.31^b^	21	
Ca	0.78 ± 0.07	8	0.63 ± 0.04	6	0.81 ± 0.11	15	0.95 ± 0.30	31	P = 0.119*
Mg	0.42 ± 0.01^ab^	2	0.37 ± 0.05^b^	14	0.47 ± 0.06^ab^	13	0.52 ± 0.09^a^	18	

	µg g^−1^	RSD%	µg g^−1^	RSD%	µg g^−1^	RSD%	µg g^−1^	RSD%	
Na	159 ± 5	3	98 ± 14	15	171 ± 87	51	311 ± 133	43	P = 0.127*
Fe	121 ± 16^a^	13	98 ± 13^a^	13	135 ± 32^a^	24	372 ± 69^b^	25	
Zn	81 ± 2	3	87 ± 11	13	113 ± 14	12	155 ± 32	21	P = 0.043*
Mn	68 ± 2^a^	3	69 ± 2^ab^	3	89 ± 12^ab^	14	110 ± 33^b^	30	
B	14 ± 2^a^	11	11 ± 1^a^	7	17 ± 3^a^	21	103 ± 24^b^	23	

Accurate and precise data was measured for the standard 100 mg plant material (Arabidopsis seeds, n = 4) and compared with the measurement of 1 mg seed material and further down to 10 seeds (n = 10) and 1 seeds (n = 10). Data was tested with a one-way ANOVA t-test, and the asterisks indicate significant differences (*P ≤ 0.05; **P ≤ 0.01; ***P ≤ 0.001) between the root tissue and the blank samples for each element (n = 10). *The differences in the median values among the treatment groups are not great enough to exclude the possibility that the difference is due to random sampling variability; there is not a statistically significant difference

Different letters (a, b) indicate significant differences between measurements

Based on the successful analysis of a batch of only 10 Arabidopsis seeds, we proceeded to analyse seed fractions, i.e. the seed coat (outer seed layers plus the endosperm) and the embryo, which had been isolated and pooled from 10 individual seeds. The seed dissection was carried out manually, with forceps. Hence each sample contained either 10 whole seeds, 10 seed coats or 10 embryos harvested from either control plants (−Zn) or plants that had been subjected to a high Zn supply (+Zn). The aggregated element contents in seed coat and embryo were then compared to the content in whole seeds (ng seed^−1^) and the recovery of each element was used as a validation of the method (Table 3). An aggregated content lower than the whole seed content (recovery < 100%) would indicate either poor digestion of the fractions and/or loss of material during dissection and handling. In contrast, an aggregated content higher than the whole seed content (recovery > 100%) would indicate contamination during handling and/or analysis. To avoid contamination and because of the small sample amounts, all data in Table 3 are presented as total element contents (ng) and not as a concentrations, since it was not possible to obtain reliable weights of the seed fractions.

**Table 3:** Elemental distribution between seed coat and embryo of wild-type Arabidopsis seeds grown with and without added Zn

Element	Whole seed ng seed^−1^		Seed coat ng seed^−1^		Embryo ng seed^−1^		Ratio Embryo/seed		Recovery %	
	−Zn	+Zn	−Zn	+Zn	−Zn	+Zn	−Zn	+Zn	−Zn	+Zn
S	1752 ± 287	1624 ± 214	422 ± 179	408 ± 152	1149 ± 199	986 ± 265	2.7	2.4	90	86*
P	1631 ± 195	1636 ± 115	226 ± 44	206 ± 42	1251 ± 190	1229 ± 142	5.5	6.0	91	88*
K	1436 ± 175	1767 ± 363	309 ± 66	203 ± 47	652 ± 110	411 ± 96	2.1	2.0	67*	35*
Mg	570 ± 76	592 ± 47	76 ± 14	99 ± 15	441 ± 76	455 ± 58	5.8	4.6	91	85*
Fe	22 ± 1	16 ± 1	4 ± 1	3 ± 1	17 ± 2	12 ± 1	4.3	4.0	95*	95
Zn	16 ± 6	51 ± 3	4 ± 1	12 ± 2	11 ± 2	31 ± 4	2.8	2.6	89	91
Mn	13 ± 3	13 ± 1	4 ± 1	4 ± 1	8 ± 2	7 ± 2	2.0	1.8	90	90
B	1.9 ± 0.7	3.1 ± 0.54	1.6 ± 0.4	1.7 ± 0.5	1.0 ± 0.3	0.8 ± 0.4	0.6	0.5	135*	79*
Cu	1.3 ± 0.2	1.2 ± 0.2	0.8 ± 0.3	0.7 ± 0.2	0.7 ± 0.3	0.4 ± 0.1	0.9	0.6	119*	91
Mo	0.38 ± 0.04	0.27 ± 0.02	0.23 ± 0.06	0.12 ± 0.03	0.28 ± 0.04	0.16 ± 0.03	1.2	1.3	134	103

Analysis was done on batches of 10 whole seeds and 10 seeds separated into seed coat and embryo fractions. Data are expressed in ng of an element ± the standard deviation (n = 10). The recovery of the individual elements was calculated as the ratio between their summed quantity (ng Zn in seed coat plus ng Zn in embryo) divided by that in the whole seed. Data was tested with a one-way ANOVA t-test, and the asterisks indicate significant differences (*P ≤ 0.05; **P ≤ 0.01; ***P ≤ 0.001) between the root tissue and the blank samples for each element (n = 10)

For the elements Mg, P, S, Fe and Zn, the recovery ranged between 85 and 108%, while for the microelements B, Cu and Mo, a larger deviation in recovery was recorded (79–135%). For K, the recovery was only 67% or 35% (+Zn and −Zn, respectively), thus indicating that the sample preparation method, which included soaking the seeds in water in order to facilitate the dissection, was not suitable for measuring K due to substantial leaching. The Zn treatment of the plants had a strong effect on the Zn content of the whole seed (51 and 18 ng in the +Zn and −Zn treatment, respectively). The ratio between the Zn content in the embryo (33 and 11 ng, +Zn and −Zn, respectively) with that in the seed coat (13 and 4 ng, +Zn and −Zn, respectively) was similar for the two treatments (2.5 and 2.75, +Zn and −Zn, respectively) (data from Table 3).

### Analysis of isolated barley root tissues

**Fig. 2:**
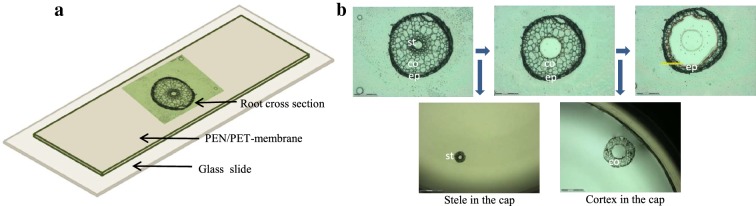
Sample collection with laser micro-dissection for ICP-MS analysis. Schematic illustration of a cross section from barley roots, mounted on a PEN or PET plastic membrane (**a**). Laser capture microdissection collection of the stele and cortex tissues from a 60 µm thick cross section of barley root (**b**). The area of the stele is about 30,000 µm^2^ and the area of the cortex is about 150,000 µm^2^, corresponding to volumes of 0.0018 and 0.0090 mm^3^, or 1.8 and 9 nL, respectively, of the root cylinder

As a final challenge of the method, isolated tissue from microscopic specimens were collected by LCM and analysed by ICP-MS. Cryo-sections (30–90 µm thick) from 17-day-old barley roots were prepared according to Persson et al. [21], and mounted on polyethylene naphthalene (PEN) membrane covered glass slides (Membrane Slide 1.0 PEN; Carl Zeiss Microscopy). Parameters for laser cutting and catapulting were optimized on pure plastic membranes, then applied to 30, 60, 90 µm thick root sections (Fig. 2). It proved to be equally feasible to cut and catapult the stele and cortex of 30 and 60 µm thick sections, whereas several laser shots were needed in order to completely cut and catapult the 90 µm thick sections. In order to get as much sample material as possible for the subsequent ICP-MS analysis, 60 µm thick sections were selected. To test the detection limit for nutrient elements, isolated stele and cortex tissues from three different cross-sections were pooled into one sample (with 4 repetitions, i.e. 24 collection events in all). The specific tissues were outlined, cut and collected using the laser pressure catapulting (LPC) function of the instrument (Fig. 2). In the first attempts, the catapulted tissue was collected into the cap of a collection tube, which had an adhesive coating on the inside. However, as the tissues adhered firmly to this surface it was difficult to transfer the samples to the vials used for micro-digestion. Hence, instead of using a vial with an adhesive cap we used ordinary PCR vials (200 µL) and filled the cap with 40 µL of Milli-Q water. This approach allowed trapping of the tissue in the water drop so that the sample transfer could be done quantitatively with a pipette. In order to correct for background signals, blank samples from the PEN membrane were analysed. The cutting area from each sampled area (either stele or cortex) was copied using the software, providing a blank sample with identical area from the plastic membrane surrounding the sample. The value of this blank sample was then subtracted from the values obtained by analysis of the tissue-containing samples.

The LCM-collected samples were very difficult to locate in the water-filled cap, especially the transparent blank collections of PEN plastic. In order to test if a marker ion for collected plant material could be obtained, the plant cultivation protocol was modified by addition of HoCl_3_ (Holmium chloride) to the growth medium in a final concentration of 1 µM. Another ion, rubidium (Rb), was added to the PEN plastic membrane, using an ordinary marker pen. Briefly, a marker pen was opened and 500 µL of an Rb standard solution (CPI International, Santa Rosa, USA) was injected into the ink-holder with a syringe and left to equilibrate for 3 h. Hereafter the ink of the pen contained Rb, which could be added to the membrane by drawing. By these preparations, a marker ion for each of the different collections [i.e. root tissue plus PEN membrane vs. PEN membrane only (i.e. blanks)] was obtained. Accordingly, both Ho and Rb signals were expected in the root tissue collections and only the Rb-signal was expected in the blanks. Indeed, ^165^Ho signals were clearly detected both in the stele (~ 2000 counts) and cortex (~ 300 000 counts) samples and served as a verification that plant material had been correctly sampled (Additional file 1). As expected, no ^165^Ho signal was detected in the blank samples. The Rb-signal, on the other hand, was detected in both sample types, with similar ion intensity, indicating that plant and blank samples had been collected, transferred, digested and finally analysed in an identical way (Additional file 1). The Ho-addition to the growth media was hence abandoned in the future plant cultivation protocol.

Measurements of the ion intensities of ^55^Mn and ^66^Zn in cortex and stele showed that these two elements were present in both root sample types, but also in the blanks (Additional file 1). Thus, significant problem with background contamination of these elements, and possibly also of other elements, were revealed. This contamination likely originated from the catalysts used during polymerization of the plastics. The test was repeated with surface-washed PEN-membranes, however with similar results (data not shown). In an attempt to avoid the background contamination from the PEN-membranes, the root sections were put directly on a normal glass slide, however, it was not possible to quantitatively collect all the tissue in this way. Hence, this approach was abandoned.

Realizing that the PEN membrane gave significant background counts for a number of our target analytes (i.e. plant nutrient elements), alternative membrane types made from polyethylene napthalate (PEN), polyethylene terephthalate (PET), polyphenylene sulphide (PPS) or polyesther (POL) were analysed by LA-ICP-MS in order to test which of the plastics that contributed least with background element signals (Table 4). The results from this analysis showed that the different membranes had very different element backgrounds. The PEN membrane had, by far, the highest background of Mn, whereas that of Zn was negligible. The previous observation of Zn contamination on this particular plastic (Additional file 1) was hence interpreted as surface contamination. The POL membrane had the highest concentrations of Fe, Zn and Cu, while the PPS membrane showed a high contribution from S. The PET membrane had the lowest concentration of the micronutrients Mn, Fe, Zn and Cu, but slightly higher background of Ca than the PEN membrane. Since we aimed to focus on the essential plant micronutrients, the PET membrane slide was chosen for the following sample preparations. To further reduce the background, the PET membrane slides were washed with milli-Q-H_2_O and then thoroughly air-blown before use. In addition, the Teflon vials (0.7 mL) and their lids as well as the polypropylene (PP) 300 µL HPLC vials, lids and pipette tips were all placed in 7% HNO_3_ overnight, then rinsed in Milli-Q-H_2_O and left to dry in a LAF bench.

**Table 4:** Ion intensity (average counts) in different plastic membranes

	Mg	P	S	K	Ca	Mn	Fe	Cu	Zn
PEN	n.d.	90 ± 20	n.d.	n.d.	n.d.	4620 ± 400	n.d.	n.d.	n.d.
POL	410 ± 30	160 ± 30	n.d.	n.d.	1960 ± 170	n.d.	1100 ± 930	20 ± 10	470 ± 90
PPS	420 ± 50	n.d.	19770 ± 560	n.d.	1970 ± 160	n.d.	n.d.	n.d.	n.d.
PET	80 ± 20	160 ± 10	n.d.	n.d.	1360 ± 340	n.d.	n.d.	n.d.	n.d.

Ion intensity (average counts) in the different plastic membranes polyethylene napthalate (PEN), polyethylene terephthalate (PET), polyphenylene sulphide (PPS) and polyester (POL). The plastic membranes were analyzed by LA-ICP-MS, and data represent the average ion intensities ± SE from a 300 × 300 µm square (n.d. = no signal detected)

**Fig. 3:**
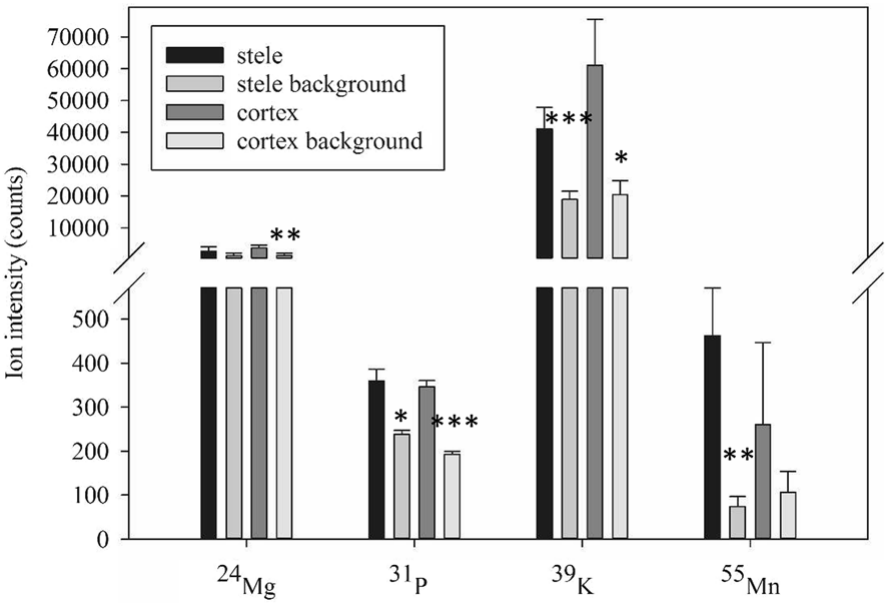
Ion intensity signals of Mg, P, K and Mn in the stele and cortex tissues of barley root and their corresponding blanks. The stele and cortex tissues were micro-dissected, collected and pooled from 3 cross-sections mounted on PET membrane-covered glass slides. Representative blank samples (with identical area as the tissue sample) were cut and collected from the membrane where no plant tissue was present. All samples were digested and analyzed by ICP-MS. Data was tested with a one-way ANOVA t-test, and the asterisks indicate significant differences (*P ≤ 0.05; **P ≤ 0.01; ***P ≤ 0.001) between the root tissue and the blank samples for each element (n = 4)

The samples of stele and cortex tissue were re-analysed together with their corresponding blanks, using the optimised procedure (Figs. 2 and 3). In particular the Mn-signals had been significantly reduced from > 120,000 counts on the PEN membranes (Additional file 1) to a couple of hundred counts on the PET membranes. Typical sample areas were ~ 30,000 µm^2^ for the stele and ~ 150,000 µm^2^ for the cortex. With a thickness of 60 µm, these areas correspond to ~ 0.0018 mm^3^ and ~ 0.0090 mm^3^, or a tissue volume of 1.8 and 9 nL, respectively. These volumes correspond to approximately 126 and 630 ng per tissue, according to the assumed tissue density (i.e. 1 g cm^−3^ of fresh root and 7% dry matter) and 378 and 1890 ng per sample, since we pooled 3 dissected tissues into one sample. Using PET-membranes, the ion intensity (counts) of Mg, P, K and Mn was now significantly higher in both of the two tissue types, compared to their respective PET-blanks. Other elements, for example Na, Zn, Ca, Cu, Fe and Mo, typically had 10–30% higher mean ion intensities in both of the root tissues than their corresponding blanks, however with a large variability, resulting in non-significant differences (Additional file 2).

**Fig. 4:**
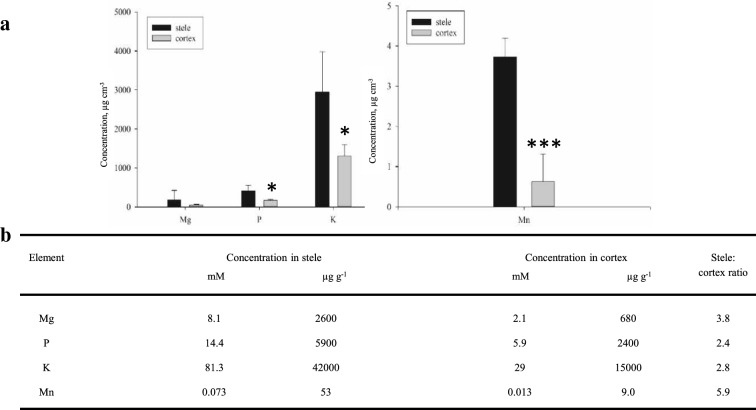
Measured concentrations (µg cm^−3^) of Mg, P, K and Mn per unit volume and the estimated concentrations of Mg, P, K and Mn per unit tissue water (mM) and per unit dry weight (µg g^−1^). Measured concentrations (µg cm^−3^) of Mg, P, K and Mn per unit volume of tissue obtained by laser micro-dissection of the stele and cortex of barley roots (**a**). Estimated concentrations of Mg, P, K and Mn per unit tissue water (mM) and per unit dry weight (µg g^−1^) of stele and cortex together with the resulting stele:cortex ratio (**b**). The concentrations were calculated assuming a fresh root tissue density of 1 g cm^−3^ and assuming a dry matter content of 7% in the roots. Stele and cortex were microdissected, collected and pooled from 3 cross-sections and subsequently digested and analyzed by ICP-MS. Data was tested with a one-way ANOVA t-test, and the asterisks indicate significant differences (*P ≤ 0.05; **P ≤ 0.01; ***P ≤ 0.001), between the tissues, for each element (n = 4)

ICP-MS analysis of the cortex and stele tissues collected by LCM showed that P, K and Mn all had significantly higher concentration in the stele than in the cortex, whereas the difference in Mg concentration was insignificant (Fig. 4). The K concentration was highest in both tissue types, with concentrations of 81.3 and 29 mM (molar concentration per unit tissue water for stele and cortex, respectively). Assuming a dry matter content of 7%, these values correspond to 42,000 µg g^−1^ (4.2%) and 15,000 µg g^−1^ (1.5%), respectively (element mass per unit dry matter). Phosphorus had the second highest concentration, followed by Mg and then Mn, both in the stele and in the cortex. The gradient between stele and cortex was steepest for Mn, showing an almost sixfold higher concentration in the stele compared to the cortex (53 and 9.0 µg g^−1^ DM in the stele and cortex, respectively) (Fig. 4). Phosphorus (P) and K had 2.4 and 2.8 times higher stele than cortex concentrations, respectively.

In order to compare the LCM-derived concentrations with results from an independent analytical technique, the relative ion intensities of Mg, P, K and Mn were measured by LA-ICP-MS analysis of a similar cross section (same plant, same root, neighbouring cross section, same thickness) (Additional file 3). This analysis showed that Mn was mainly accumulating in and around the stele, but also in the outermost cell layer, i.e. the epidermis, which was not included in the LCM-based analysis. The ^55^Mn ion intensity signals (counts) from the stele and cortex were extracted from the Mn-image, as displayed in Additional file 4. The ratio between the average counts from the stele and cortex was approximately 5.3 (677/127), i.e. similar to the corresponding ratio from the LCM-based analysis.

## Discussion

Ionomic analyses at the cellular or tissue level is to date challenged by the small sample sizes (nanogram-sized) and the difficulties in obtaining pure samples containing identical cell types reflecting the true element composition. Indeed, multi-element profiling at the cellular level is possible with SC-ICP-MS techniques, which can introduce, detect and quantify the element content in individual cells. Together with the extraordinary sensitivity and multi-element capacity of ICP-MS, absolute quantification of most elements of the periodic table is possible, typically presenting concentrations in quantity of element per cell [12]. Such analyses, however, require isolated cells/cell cultures, which make a vast majority of plant samples out of reach for multi-element/ionomic profiling. In some cases pure plant tissues can be collected, e.g. epidermal bladder cells [24] and pollen cells [8], but special methods are required to sample cells that are not immediately accessible. Specific plant tissue or specialized plant cells (i.e. endodermis, cortex or epidermis cells) can be sorted and isolated by e.g. FACS for subsequent analysis [14, 25]. However, in plants this kind of sorting requires that the different cell types express specific fluorochrome markers, which to date is only available in mutants of Arabidopsis. Moreover, both the sorting and analysis of such samples induce a risk of ion leakage or displacement, especially for monovalent ions that do not form covalent bonds (e.g. K^+^ and Na^+^) [21].

Instead of isolation and collection, specific tissues or individual cells can be analysed in situ by various element bioimaging techniques [2]. These techniques are, however, rarely ideal for quantitative ionomic analyses, since absolute quantification requires that calibrations standards are available with the same matrix composition, micro-topography and hardness as the sample to be analysed. In biological samples, this is very difficult to obtain, rendering these techniques as merely semi-quantitative [26]. Other disadvantages with respect to tissue-specific ionomics are higher detection limits (compared to conventional ICP-MS analysis) and limitations in the range of elements that can be analysed simultaneously [2, 21, 22].

In this study we demonstrate that both collection and quantitative ICP-MS analyses of nanogram-sized samples is possible without compromising data quality. As such, our method constitutes a novel tool for tissue-specific ionomic profiling at the cellular level. The keys to these successful method developments were the use of LCM in combination with micro-scaled pressurized digestion, an optimized low-flow sample introduction system as well as identification and reduction of contamination sources.

As a first step in the downscaling of sample size, we minimized the required sample amounts and the final volume of the samples based on analysis of certified reference material consisting of apple leaves. For this purpose, a pressurized chamber digestion system was used, in which a uniform pressure is applied to all samples, providing efficient digestion without boiling. This technique is different from closed systems with one bomb per sample, which require identical sample amounts and digestion volumes in all bombs in order to obtain the same partial pressure. The closed systems accordingly have poorer reproducibility, both with respect to samples in the same batch and between batches. The flexibility is also lower, since all samples need to have a fixed ratio between sample amount and the volume of digestion solution. In the pressurized chamber system, however, both sample amount and volume of digestion solution can be individually adjusted without compromising digestion efficiency and reproducibility.

We successfully analysed CRM material down to a sample quantity of 1000 µg (1 mg). However, for smaller samples (e.g. 500 µg) both the accuracy and precision became poorer, which we assumed was mainly due to less exact determinations of sample weights (Table 1). Hence, other strategies were required to validate obtained data for samples < 500 µg. One such strategy was to compare the analysis of Arabidopsis seeds, both as large, weighed batches (10 and 100 mg corresponding to approx. 50 and 5000 seeds, respectively), batches of 10 seeds (~ 200 µg) and single seeds (< 20 µg). In the analysis of single seeds, 5 out of 10 elements deviated significantly from the estimated concentration per seed in the 100 mg batch (Table 2). This variability, probably mostly of biological origin, were evened out in a batch of only 10 seeds, where in fact no significant differences in element concentrations were found relative to the larger samples (Table 2). The method was further challenged by investigating the element concentration in manually dissected seed fractions from Arabidopsis. Apart from K, which had a very low recovery, other elements had recoveries between 85 and 108%, indicating that it was possible to successfully digest and analyse such samples (< 20 µg) without losing material or contaminating the samples. Potassium (K) is known to be a highly leachable ion [21], and a substantial amount of this element was apparently lost during soaking of the seeds, which was a preparation step required in order to enable the manual dissection (Table 3).

For further downscaling, LCM was used to isolate tissues from barley roots. Analysis of isolated root tissues requires that both the cellular structures and the native ionic composition in the cells are unaltered, during both sample preparation and analysis. We have previously developed a sample preparation method, which encapsulates the fresh tissue with paraffin prior to freezing and cryo-sectioning. This is an essential step to avoid the displacement of elements and limit ion leakage [21]. Moreover, the choice of plastic membrane turned out to be very important for maintaining a low element background. Apparently, different metal-containing catalysts are used in the polymerization processes, which affect the element background. Hence, depending on the target analytes of a specific analysis, the choice of plastic membrane has to be carefully assessed.

In the barley root tissue fractions, potassium (K), manganese (Mn), phosphorus (P) and magnesium (Mg), were all successfully quantified in the two distinct tissue types collected (cortex and stele), which made it possible to establish inter-tissue concentration gradients of these elements, inside the root. The analysis showed that the K concentration was clearly highest and Mn was the lowest, in both tissue types. It is well known that K concentrations in any plant material are substantially higher than both P, Mg and Mn. In whole roots, reported K concentrations in the literature range from 2 to 6% element concentration per unit dry matter, where < 2% represent deficient conditions and 6% represent “luxury consumption” [27, 28]. Our results were between 1.5 and 4.2%, in cortex and stele, respectively, hence showing a similar concentration range, but also revealing large, tissue-specific concentration differences. Other reported K concentrations in barley roots typically range from 71 to 119 mM [29] and 75–83 mM [30], using micro-electrodes. These values should rather be compared to our mM values, since they reflect fresh tissue measurements. Again, our values (29 and 81.3 mM in cortex and stele, respectively) were in a similar concentration range, yet with large inter-cellular differences (Fig. 4). Similar differences in K distribution were measured more recently, showing an approximately threefold higher K concentration in the stele compared to the cortex [31], which again is comparable to our measurements (K-ratio; stele: cortex = 2.8, Fig. 4). The reported average cytosolic P concentrations in maize root segments were reported to be 6.5 µmol cm^−3^ [32]. Hence, also for P, our measurements seem plausible [measured values 416 µg cm^−3^ (= 13.4 µmol cm^−3^, and 14.4 per unit tissue water (mM)) and 171 µg cm^−3^ (= 5.5 µmol cm^−3^, and 5.9 per unit tissue water (mM)), in stele and cortex, respectively (Fig. 4)].

In order to further validate and compare our results, barley root samples from the same batch used for LCM collection were also analysed by LA-ICP-MS, where the raw signals were assumed to be proportional to the concentration. For Mn, the raw signals (counts) were extracted from the image (Additional file 3), generating a stele: cortex ratio of approx. 5.3 (Additional file 4), which was comparable to the ratio of 5.9 obtained in the LCM analysis combined with ICP-MS (Fig. 4).

Further reduction of background contamination of a number of elements is required in order to establish a true multi-element analysis of micro-dissected root tissues (Additional file 2). This would probably require cleaner laboratory environments for sample collection and handling, as well as cleaner vials, chemicals and plastic tubings. A major benefit with LCM in this context, however, is the possibility of pooling samples in order to increase the number of detectable elements. A long analysis time per sample will permit the full power of ICP-MS detection to be utilized, since then the same identical sample can be analysed in different modes (e.g. collision/He, reaction/H_2_ and O_2_-mode), all which offer optimum sensitivities for different sets of elements. With a flow-rate of only ~ 50 µL min^−1^, and a sample volume of 300 µL, our method permits at least 4 min of analysis, if needed (4 min × 50 µL min^−1^ = 200 µL of sample consumed). Four minutes is enough to analyse the same sample in all of the different gas modes. These possibilities were not fully explored in this study, but we anticipate that this could further increase the number of elements to be included in the analysis [e.g. S and Arsenic (As) in oxygen mode, Fe and Se in H_2_ mode].

Concentration gradients between the stele and the surrounding cortex will indicate how efficiently mineral nutrients are transported towards the xylem, for further transport to the shoot. If various parts of the same identical root is sampled, such ratios may also indicate which part of the root that is responsible for nutrient transport and how efficient the transport to the shoot is. Furthermore, the presented method has a unique potential for being combined with other ‘omics’-techniques, e.g. transcriptomics, proteomics and metabolomics, which are already established techniques, both in combination with FACS and LCM [24, 25, 33, 34]. Such combined analyses have the potential to shed light on the genetic mechanisms that regulate the plant ionome on the cellular level, which is pivotal knowledge for advancing our understanding of e.g. nutrient use efficiency, environmental monitoring, evolutionary biology and biofortification.

## Conclusions

The method presented here allows accurate and reproducible collection, digestion and ICP-MS analysis of specific plant tissues in the nanogram-size range. As such, the method provides a novel tool for precise tissue isolation, absolute quantification of elements and ionomic profiling on an unprecedentedly small scale. In combination with already existing methods for transcriptomic, proteomic and metabolomic profiling, ionomic studies at this level of detail have a unique potential to forward our understanding of the processes involved in regulating nutrient homeostasis and nutrient use efficiency.

## Materials and methods

### Sample material

The certified reference material (CRM) NIST 1515 (apple leaf, particle size < 75 µm), was purchased from the US Department of Commerce, National Institute of Standard and Technology (Gaithersburg, MD, USA). In the single seed and seed fraction analyses of *Arabidopsis thaliana*, wild-type seeds were always used (Col-0). The seeds were harvested from plants growing in soil under long-day conditions in controlled growth chambers (16 h light at 120 µmol m^−2^ s^−1^, 22 °C and 8 h dark at 20 °C, 70% humidity). Plants for the Zn experiment were watered twice a week with either pure water or a 3 mM ZnSO_4_ solution. In order to analyse the distribution of elements, Arabidopsis seeds were manually dissected into two parts, representing the seed coat fraction (with the endosperm still attached) and the embryo fraction. Seeds were rinsed in Milli-Q Element water (Millipore Corporation, DK) and soaked on wet filter paper for 2 h prior to dissection, using forceps under a stereomicroscope.

The 17-day-old barley (*Hordeum vulgare* L., cv. Irina) plants used for root analyses were germinated in vermiculite for 7 day and then cultivated at 16 h day length and 18 °C/15 °C day/night temperature cycles in a greenhouse. Uniform seedlings were transferred to light-impermeable black 5 L cultivation units, each unit holding 3 plants. The units were filled with a nutrient solution containing 200 µM KH_2_PO_4_, 200 µM K_2_SO_4_, 300 µM MgSO_4_·7H_2_O, 100 µM NaCl, 300 µM Mg(NO_3_)_2_·6H_2_O, 900 µM Ca(NO_3_)_2_·4H_2_O, 600 µM KNO_3_, 50 µM Fe(III)-EDTANa, 0.8 µM Na_2_MoO_4_·2H_2_O, 0.7 µM ZnCl_2_, 0.8 µM CuSO_4_·5H_2_O, 0.8 µM NiCl_2_, and 2 µM H_3_BO_3_. The solutions were replaced once per week and the pH was adjusted daily with HCl to 6.0 ± 0.2.

### Sample digestion

The apple leaf CRM was weighed in amounts ranging from 500 µg to 2000 µg on a high-accuracy balance (MT5, Mettler-Toledo International, Columbus, USA) and transferred to commercially available 0.7 mL, 12 × 32 mm Teflon vials (Chromacol 0.7 CTVT, VWR, Denmark). A digestion solution with a 2:1:1 ratio of 67% HNO_3_ (PlasmaPURE; SCP science, Quebec, Canada), 30% H_2_O_2_ (Analytical grade 31,642, SigmaAldrich, St. Louis, USA) and MilliQ–element water was added to the digestion tubes, providing a total volume of 50 µL digestion solution. The tubes were then placed in a 22-position sample rack, traditionally used for pressurized digestion of samples ranging from 20 to 500 mg. As the original vials were 8 cm tall, an in-house designed Teflon-lid with loose fit ensured stability of the 3 cm vials now standing in the rack. This lid avoided water condensation into the vials after digestion (Fig. 1).

All samples (i.e. CRM, Arabidopsis seeds/seed fractions and LCM-collected samples) were digested in a pressurized digestion chamber (Ultrawave, Milestone Inc., Bergamo, Italy) at 240 °C for 10 min with a reduced amount of ballast water for heat transfer. The full cycle consisted of 15 min ramping to the digestion temperature, maintenance of this temperature for 10 min and then cooling down for another 15 min, giving a total runtime of 40 min. The 240 °C temperature ensured a complete digestion of the samples, hence decreased the residual carbon to a minimum [22]. After digestion, samples were transferred quantitatively to polypropylene HPLC-vials and diluted with Milli-Q water to a final acid concentration of 3.5%, which resulted in a final volume of ~ 300 µL (300 mg).

### Multi-elemental analysis

The elemental analyses were done with an ICP-MS (7900, Agilent Technologies, Manchester, UK) equipped with a Micromist nebulizer with ratchet gas fittings (Agilent Technologies, Manchester, UK). The peristatic pump on the ICP-MS was equipped with TYGON tubings with an inner diameter of 0.19 mm (TYGON R3607 0.19 I.D., Agilent Technologies, Manchester, UK), generating a flow of 50 µL min^−1^. The system was rinsed with 3.5% HNO_3_ between every sample. Samples were injected by use of an autosampler (I-AS, Agilent Technologies, Manchester, UK) with inserts to fit the 300 µL HPLC vials. External calibration was made using a custom-made, non-equimolar multi-element standard (P/N 4400-ICP-MSCS, CPI International, Santa Rosa, USA) which corresponds to concentrations typically found in plants. The ICP-MS instrument was operated in collision mode, using helium (He) as collision gas with a flowrate of 4.0 mL min^−1^.

### Sample preparation for LCM collection and LA-ICP-MS analysis

Sample preparation followed the same procedures as described by Persson et al. [21]. Uniform seminal roots of 17-day-old barley plants were selected and cut 2 cm behind the root tip of the main axis. Briefly, 1 cm root pieces were encapsulated in molten paraffin, then embedded in OCT (Optimal Cutting Temperature; Tissue-Tek, Sakura Finetek, Tokyo, Japan) and instantly frozen in liquid nitrogen (N_2_). After freezing, 30–90 µm thick cross-sections were cut with a cryotome (Leica CM050S, St. Gallen, Switzerland), and transferred onto membrane-covered glass slides (Carl Zeiss MicroImaging GmbH, Jena, Germany). Sections for LA-ICP-MS analysis were prepared similarly, but instead transferred to plastic microscopy slides (Permanox Microscope Slide, Electron Microscopy Sciences, Hatfield, USA). The mounted sections were then allowed to slowly freeze-dry overnight (− 25 °C, ~ 16 h) inside the cryotome.

Optimal laser settings for LCM-dissection (Laser Micro-Dissection (LCM), Carl Zeiss MicroImaging GmbH, Jena, Germany), was achieved using the following settings (% of max; 100%): cut speed 10–20; cut energy 48–53; cut focus 75–80; laser pressure catapulting (LPC) energy: 68–73; LPC focus: 75–80. Prior to tissue collection with LCM, the cap of a 200 µL PCR tube (VWR International, LLC Radnor, USA) was filled with 40 µL Milli-Q water and mounted in the cap holder. Stele and cortex tissues were collected into the cap by LPC and then quantitatively transferred to digestion vials with a pipette. A headband-illuminating magnifier was used while pipetting, which helped to localize the small samples during the sample transfer. The stele samples were cut just outside of the endodermis, which is the cell layer surrounding the vascular tissues, hence endodermal cells were included in the stele samples and not in the cortex samples. The cortex samples were cut without the outermost cell layer, the epidermis, which is a functionally different tissue type (Fig. 2). Representative blank samples were collected from the plastic membrane, always using the same sample area as for the corresponding root sample. The following plastic membranes were tested: polyethylene napthalate (PEN) membrane, polyethylene terephthalate (PET) membrane, polyphenylene sulphide (PPS) membrane and polyester (POL) membrane (all from Carl Zeiss MicroImaging GmbH, Jena, Germany).

Since the area of each of the micro-dissected stele and cortex sections was known, as well as their thickness (60 µm), the volume of each sample could be calculated. After ICP-MS analysis, a concentration unit of µg cm^−3^ was thus obtained for each element. Assuming a fresh tissue density of 1 g cm^−3^ and 93% water, the µg cm^−3^ unit was converted to a molar concentration per unit tissue water (mM). The element concentration per unit dry weight (µg g^−1^ DW) was estimated assuming a dry matter content of 7% in roots of hydroponically grown barley plants (data from an in-house database).

### LA-ICP-MS analysis

LA-ICP-MS analyses of root cross sections were performed as previously described [21].

## Supplementary information

**Additional file 1.** Ion intensity of Holmium (Ho), Rubidium (Rb), Mn and Zn in stele and cortex tissues of barley roots. Ion intensity of Holmium (Ho), Rubidium (Rb), Mn and Zn in stele and cortex tissues of barley roots and PEN membrane blanks, analyzed as the isotopes ^165^Ho, ^85^Rb, ^55^Mn and ^66^Zn, respectively. Stele and cortex tissues were micro-dissected and pooled into one sample from 3 neighboring cross-sections. Representative blank samples (with identical area as the tissue samples) were cut and captured from the PEN membrane where no plant tissue was present. The tissue samples and blanks samples were digested and then analyzed by ICP-MS.

**Additional file 2.** Signal intensities of different elements in stele and cortex tissues of barley root and PET membrane blanks. Stele and cortex were collected and pooled from 3 cross-sections with laser capture microdissection. Representative blank samples (with identical area as the tissue sample) were cut and captured from the PET membrane where no plant tissue was present. The tissue samples and blanks samples were digested by and then analyzed by ICP-MS. Data was tested with a one-way ANOVA *t* test, and the asterisks indicate significant differences (*P ≤ 0.05; **P ≤ 0.01; ***P ≤ 0.001), between the root tissue and the blank samples for each element (n = 4).

**Additional file 3.** Element distribution in a barley cross section. Element distribution in a barley cross section 2 cm behind the root tip, analysed by Laser Ablation-ICP-MS. The signal intensities are displayed as heat maps where red represent the strongest intensities and purple the weakest (= background). All ion intensities were normalized to endogenous carbon (measured as ^13^C). The images are showing the ^31^P (upper left), ^55^Mn (upper right), ^24^Mg (lower left) and ^39^K (lower right) results in the same cross section (st = stele, co = cortex, ep = epidermis). The scale bars represent 50 µm.

**Additional file 4.** Manganese distribution in a barley cross section. Manganese distribution in a barley cross section, 2 cm behind the root tip, analysed by Laser Ablation-ICP-MS as ^55^Mn. The signal intensity is displayed as a heat map (upper image) where red represent the strongest intensities and purple the weakest. The signals in the graph below, stem from five transversal lines extracted from the heat map data (dotted line), showing average signals ± SE from the left side of cortex (minus epidermis), stele and right side of cortex (minus epidermis) (st = stele, co = cortex, ep = epidermis).

## Data Availability

The datasets used and/or analyzed during the current study are available from the corresponding author on reasonable request.
