# Supplementary material for: Towards single-cell ionomics: a novel micro-scaled method for multi-element analysis of nanogram-sized biological samples
Source: Plant Methods. 2020 Mar 6;16:31. doi: 10.1186/s13007-020-00566-9 (PMC7059671; doi:10.1186/s13007-020-00566-9)
Supplement: Supplementary file 1 — Additional file 1. Ion intensity of Holmium (Ho), Rubidium (Rb), Mn and Zn in stele and cortex tissues of barley roots. Ion intensity of Holmium (Ho), Rubidium (Rb), Mn and Zn in stele and cortex tissues of barley roots and PEN membrane blanks, analyzed as the isotopes 165Ho, 85Rb, 55Mn and 66Zn, respectively. Stele and cortex tissues were micro-dissected and pooled into one sample from 3 neighboring cross-sections. Representative blank samples (with identical area as the tissue samples) were cut and captured from the PEN membrane where no plant tissue was present. The tissue samples and blanks samples were digested and then analyzed by ICP-MS. [file 13007_2020_566_MOESM1_ESM.pdf]

Additional file 1.

	^{165}Ho	^{85}Rb	^{55}Mn	^{66}Zn
Stele	2040	193920	128720	1432500
Cortex	303410	142030	183280	239700
Blank stele	4	141300	85350	300100
Blank cortex	2	1136700	140100	305580
Blank HNO_3	2	30	77750	350
Blank Milli-Q-H_2O	1	120	62740	41890
