# Supplementary figures and images for: Towards single-cell ionomics: a novel micro-scaled method for multi-element analysis of nanogram-sized biological samples
Source: Plant Methods. 2020 Mar 6;16:31. doi: 10.1186/s13007-020-00566-9 (PMC7059671; doi:10.1186/s13007-020-00566-9)

## Additional file 2.

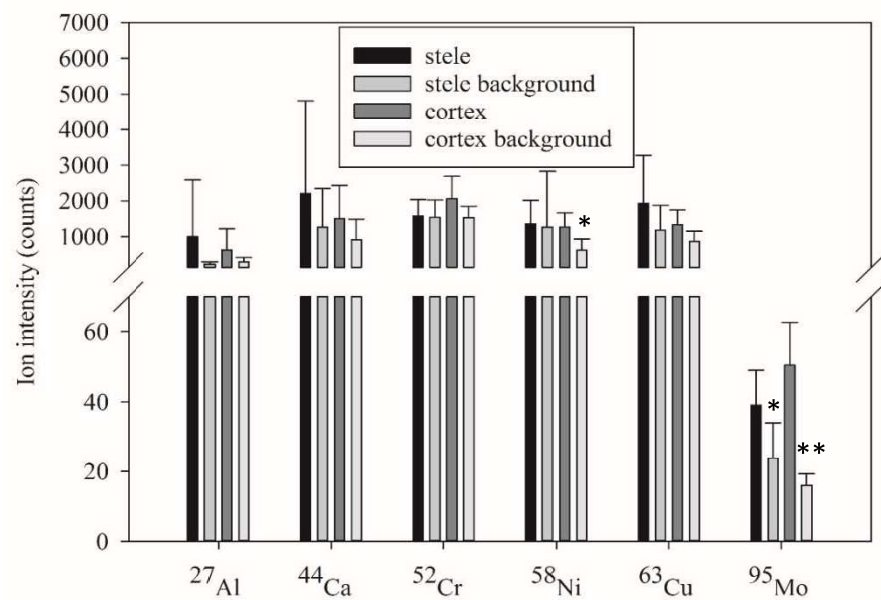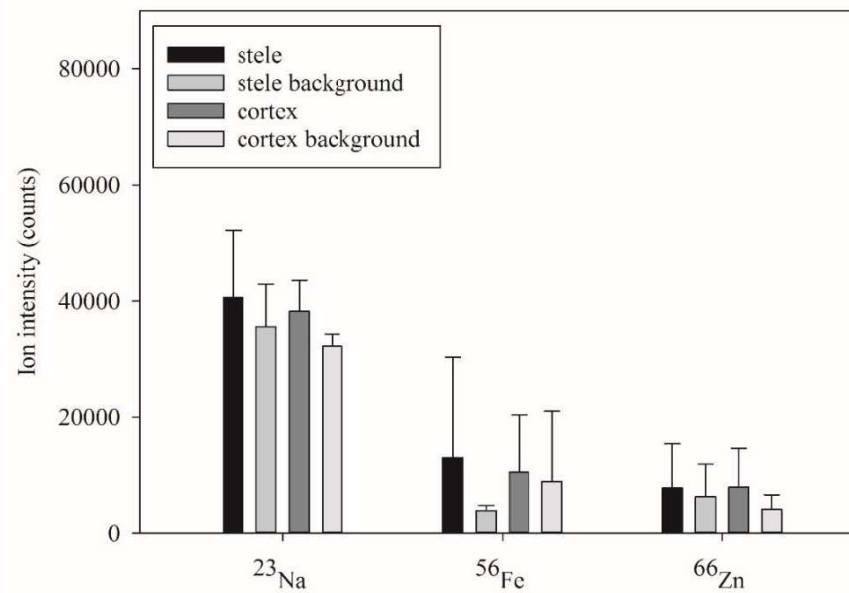

Supplement: Supplementary file 2 — Additional file 2. Signal intensities of different elements in stele and cortex tissues of barley root and PET membrane blanks. Stele and cortex were collected and pooled from 3 cross-sections with laser capture microdissection. Representative blank samples (with identical area as the tissue sample) were cut and captured from the PET membrane where no plant tissue was present. The tissue samples and blanks samples were digested by and then analyzed by ICP-MS. Data was tested with a one-way ANOVA t test, and the asterisks indicate significant differences (*P ≤ 0.05; **P ≤ 0.01; ***P ≤ 0.001), between the root tissue and the blank samples for each element (n = 4). [file 13007_2020_566_MOESM2_ESM.pdf]

Additional file 3.

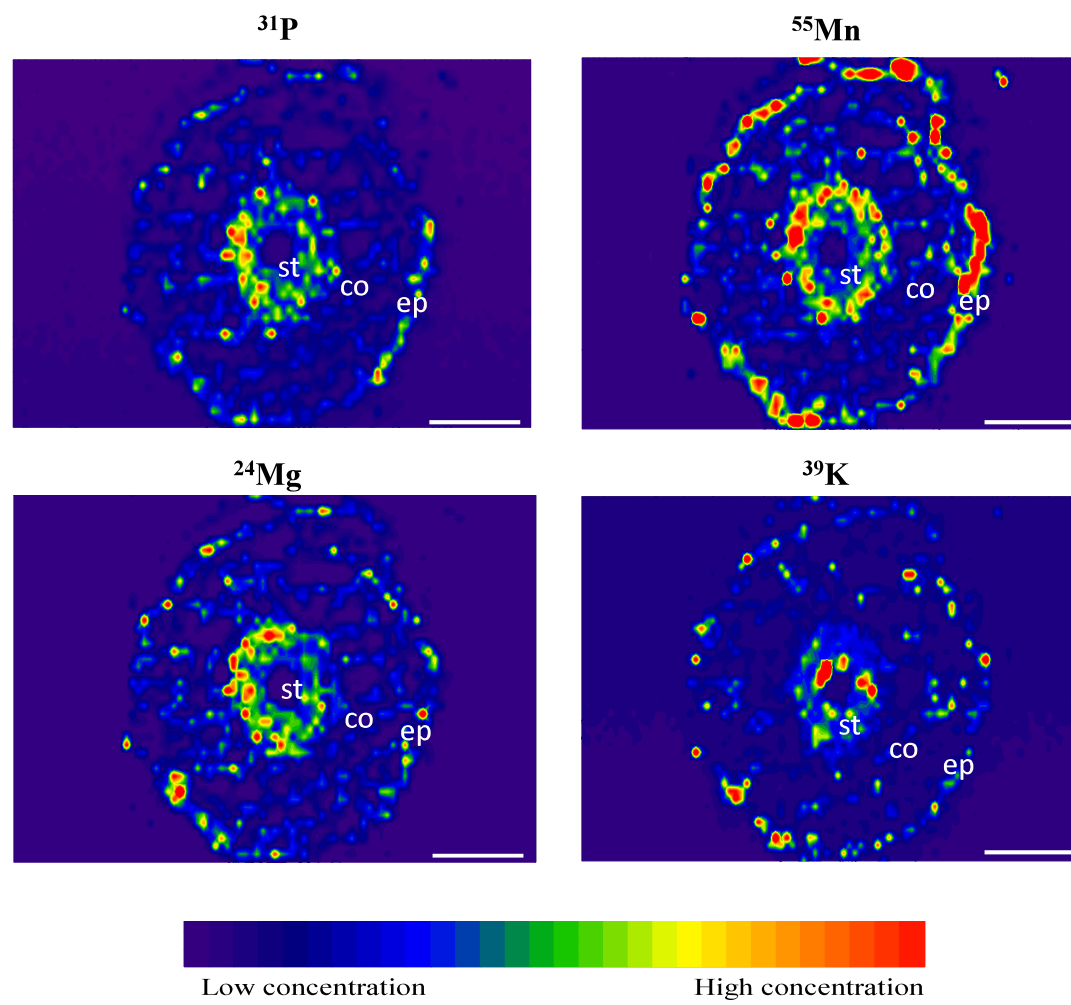

Supplement: Supplementary file 3 — Additional file 3. Element distribution in a barley cross section. Element distribution in a barley cross section 2 cm behind the root tip, analysed by Laser Ablation-ICP-MS. The signal intensities are displayed as heat maps where red represent the strongest intensities and purple the weakest (= background). All ion intensities were normalized to endogenous carbon (measured as 13C). The images are showing the 31P (upper left), 55Mn (upper right), 24Mg (lower left) and 39K (lower right) results in the same cross section (st = stele, co = cortex, ep = epidermis). The scale bars represent 50 µm. [file 13007_2020_566_MOESM3_ESM.pdf]

**Additional file 4.**

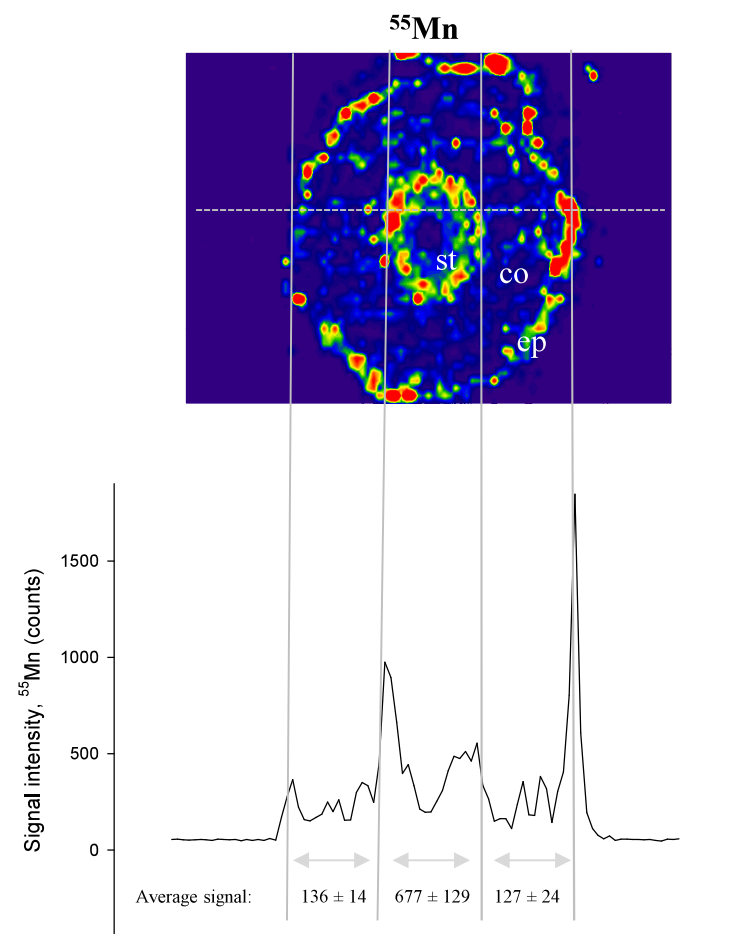

Supplement: Supplementary file 4 — Additional file 4. Manganese distribution in a barley cross section. Manganese distribution in a barley cross section, 2 cm behind the root tip, analysed by Laser Ablation-ICP-MS as 55Mn. The signal intensity is displayed as a heat map (upper image) where red represent the strongest intensities and purple the weakest. The signals in the graph below, stem from five transversal lines extracted from the heat map data (dotted line), showing average signals ± SE from the left side of cortex (minus epidermis), stele and right side of cortex (minus epidermis) (st = stele, co = cortex, ep = epidermis). [file 13007_2020_566_MOESM4_ESM.pdf]
